# Bioactivities Derived from Dry-Cured Ham Peptides: A Review

**DOI:** 10.3390/antiox14081011

**Published:** 2025-08-18

**Authors:** Noelia Hernández Correas, Andrea M. Liceaga, Adela Abellán, Beatriz Muñoz-Rosique, Luis Tejada

**Affiliations:** 1 Faculty of Pharmacy and Nutrition, Universidad Católica de Murcia—UCAM, Campus de los Jerónimos, 30107 Murcia, Spain; 2 Protein Chemistry and Bioactive Peptides Laboratory, Department of Food Science, Purdue University, 745 Agricultural Mall Drive, West Lafayette, IN 47907-2009, USA; 3 Quality and Research & Development Department, AromaIbérica Serrana, S.L. Ctra. Fuente Alamo, Km 17.4, 30332 Murcia, Spain

**Keywords:** bioactive peptides, dry-cured ham, proteolysis, functional foods, ACE-inhibitory activity, antioxidant activity, DPP-IV inhibitor

## Abstract

Dry-cured ham is a traditional food in the Mediterranean diet, which, in addition to its sensory qualities, is a natural source of bioactive peptides generated during the curing process through the action of endogenous enzymes on muscle and sarcoplasmic proteins. These low-molecular-weight peptides have attracted growing interest due to their multiple bioactivities, including antihypertensive, antioxidant, antimicrobial, antidiabetic, and anti-inflammatory effects described in vitro, in vivo, and in preliminary human studies. The identification of specific sequences, such as AAPLAP, KPVAAP, and KAAAAP (ACE inhibitors), SNAAC and GKFNV (antioxidants), RHGYM (antimicrobial), and AEEEYPDL and LGVGG (dipeptidyl peptidase-IV and α-glucosidase inhibitors), has been possible thanks to the use of peptidomics techniques, tandem mass spectrometry, and bioinformatics tools that allow their activity to be characterized, their digestive stability to be predicted, and their bioavailability to be evaluated. This review article summarizes current knowledge on the bioactivities of peptides derived from dry-cured ham, advances in their functional characterization, and challenges associated with their application in functional foods and nutraceuticals, with the aim of providing a comprehensive overview of their potential in health promotion and chronic disease prevention.

## 1. Introduction

The Mediterranean culinary tradition is characterized by numerous products, particularly dry-cured ham, especially in countries such as Spain where its consumption is deeply rooted in the food culture [1]. In addition to its sensory value, derived from its characteristic texture, aroma, and flavor, dry-cured ham is a food of great interest from a nutritional and functional standpoint. During the curing process, complex biochemical transformations take place that modify the structure and composition of the muscle [2,3], generating low-molecular-weight compounds, such as free amino acids and peptides, some of which have bioactive properties with the potential to promote health and prevent disease [4].

The ham curing process involves intense and prolonged proteolysis [3,5], in which endogenous meat enzymes (calpains, cathepsins, and various peptidases) are capable of progressively degrading the myofibrillar and sarcoplasmic proteins of the muscle [6,7,8], leading to the release of numerous peptides of various sizes and sequences. These peptides, in many cases, have relevant bioactivities documented by in vitro studies, in animal models, and, more recently, in preliminary clinical trials [4,9,10]. Among the most notable bioactivities are the antihypertensive effect, which is mediated by the inhibition of the angiotensin-converting enzyme (ACE) [11]; antioxidant activity, with the ability to neutralize free radicals and protect against oxidative damage [12]; antidiabetic action, linked to the inhibition of dipeptidyl peptidase-IV (DPP-IV) [13] and α-glucosidase [14]; antimicrobial activity against food pathogens such as Listeria monocytogenes [15]; and anti-inflammatory activity, related to the modulation of immune response mediators [16].

The growing interest in bioactive peptides in dry-cured ham has led to the development and application of advanced identification and characterization methodologies, such as peptidomics [17], tandem mass spectrometry (MALDI-TOF) [18], and the use of bioinformatic tools (e.g., PeptideRanker, BIOPEP-UWM, and CPPpred) [4,19] that allow us to predict, simulate, and study their bioactivity, stability, and bioavailability. These tools, together with simulated gastrointestinal digestion studies and absorption studies using cellular models, help clarify the fate and physiological impact of the peptides generated during curing. Taken together, these advances suggest that dry-cured ham can be considered not only a food of gastronomic interest, but also a matrix with functional and nutraceutical potential, whose applications could contribute to the prevention of chronic non-communicable diseases, such as high blood pressure, type-2 diabetes, and certain inflammatory processes.

**Figure 1:**
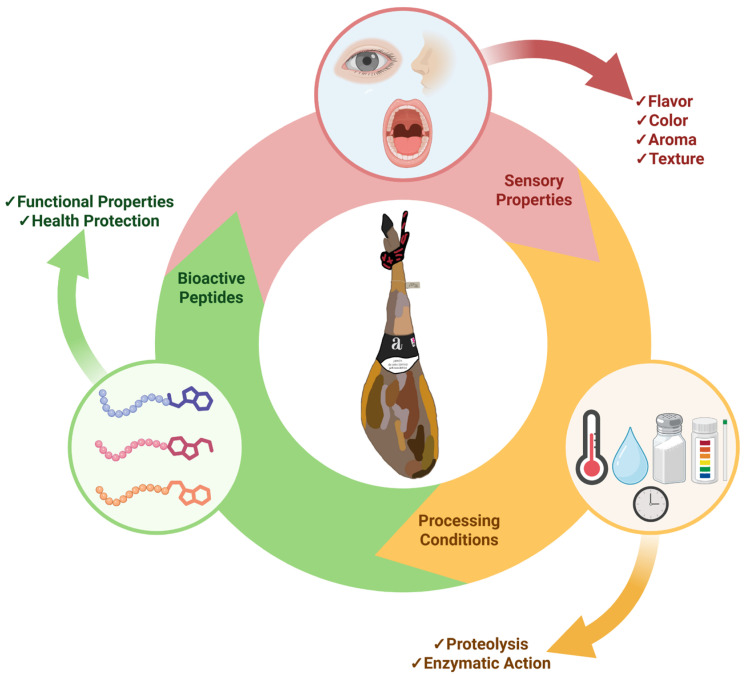
Properties of dry-cured ham. Processing conditions that affect palatability and bioactive profile. Created with BioRender.com (accessed on 1 July 2025).

This review aims to integrate and analyze current knowledge on bioactive peptides derived from dry-cured ham, with special emphasis on the biological activities described and corroborated, the mechanisms of action, and potential application in functional foods and nutraceutical products for health promotion and chronic disease prevention. The summary of the article can be seen in Figure 1.

## 2. Peptide Generation in Dry-Cured Ham

The generation of bioactive peptides in dry-cured ham is the result of a complex process of proteolysis that occurs during the curing process [20,21,22], which can last from 7 to more than 36 months (depending on the type of ham and the technological practices applied), under the combined action of a set of endogenous enzymes and, to a lesser extent, the action of microorganisms present naturally or incorporated as starter cultures in some preparations.

Calpains, calcium-dependent neutral proteases (calpaimin-1 and calpaimin-2), participate in the early stages of maturation [23,24,25], degrading structural proteins such as myosin, titin, desmin, and troponin T, and generating larger polypeptide fragments. Cathepsins, notably isoforms B, L, H, and D, are responsible for the proteolysis of myofibrillar and sarcoplasmic proteins under acidic pH and high-salinity conditions [25,26,27,28], acting both as endopeptidases and, in the case of cathepsin B, with a carboxypeptidase function. As the process progresses, exopeptidases (aminopeptidases, carboxypeptidases, and dipeptidyl and tripeptidyl peptidases) come to the fore, whose sequential action on the fragments generated by endopeptidases leads to the release of di- and tripeptides, as well as free amino acids that not only contribute to the development of the characteristic flavor of dry-cured ham [7,29,30,31], but also have significant bioactive activities in many cases [32]. This process begins after the animal is slaughtered and continues throughout all stages of curing, from salting to drying and subsequent maturation in the cellar. The peptides generated vary in size, charge, sequence, and hydrophobic/hydrophilic properties, which depend not only on the initial protein substrate and the specificity of the enzymes involved, but also on the technological conditions of the curing process. Factors that modulate the proteolysis profile, the action of enzymes, and, therefore, the generation of peptides include the curing time [33,34], which in quality dry-cured hams can exceed 30 months; the temperature and relative humidity of the maturation chambers [35]; the type and concentration of salt used; the pH of the piece; and water activity (aw) [36,37,38]. Together, these parameters determine the residual activity of proteolytic enzymes and the rate of muscle protein degradation.

The proteolysis pattern is highly specific and results in the generation of recurrent peptide sequences, many of which have been identified as bioactive [4]. For example, peptides derived from myosin (such as FPPDVGGNVD) or creatine kinase (such as AEEEYPDL) have been identified in dry-cured hams using tandem mass spectrometry techniques after fractionation by size exclusion chromatography (SEC) and reverse-phase high-performance liquid chromatography (RP-HPLC) [10,12,39]. Peptidomics studies have shown that the molecular weight of peptides with the highest bioactivity ranges from 400 to 2500 Da, concentrated mainly in the most polar fractions of the soluble extract [4,10,40].

**Figure 2:**
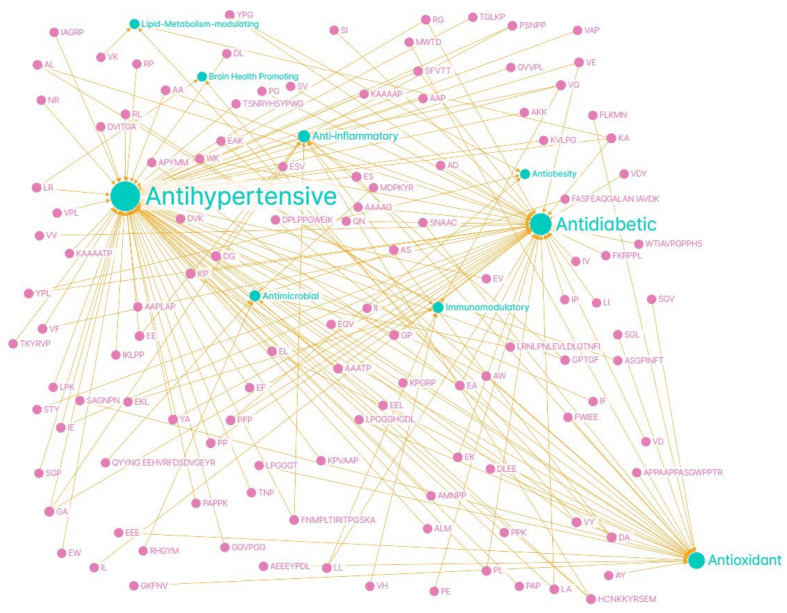
Network map summarizing the peptides identified in Spanish dry-cured ham (pink) and their associated bioactivities (green). Graph created with Graph Commons.

In addition, the peptide profile can be used as a marker of the degree of maturation and quality of dry-cured ham, and specific peptides derived from proteins such as titin or LIM domain-binding protein-3 have been identified, which can serve as biomarkers of minimum curing time and product authenticity [41]. In short, the generation of bioactive peptides in dry-cured ham is a dynamic phenomenon that is highly dependent on processing conditions and the proteolytic metabolism of the muscle itself, with direct implications for the functional value of the food. Thus, the natural proteolysis of dry-cured ham makes this food a matrix of interest for the development of functional and nutraceutical products. In the Figure 2 it could be seen a network map summarizing the peptides identified in Spanish dry-cured ham and their associated bioactivities.

## 3. Bioactivity Associated with Peptides Derived from Dry-Cured Ham

The peptides that are naturally liberated during the dry-cured ham maturation process not only participate in the development of the product’s sensory attributes, such as color, aroma, flavor, and texture [32], but also represent a source of bioactive compounds with potential beneficial effects for human health [4], as demonstrated by various in vitro, in vivo, and, increasingly, preliminary clinical trials [42,43,44].

The diversity of biological activities described for these peptides is a consequence of the variability of their sequences, sizes, and physicochemical properties, the most relevant being antihypertensive, antioxidant, antidiabetic, antimicrobial, and anti-inflammatory, among others. Among the peptides with multifunctional effects, numerous small di- and tripeptides stand out, produced by the sequential action of exopeptidases, which have the ability to act simultaneously on different physiological targets, such as the VD, WK, IE, and SI fragments, identified from proteins such as beta-enolase and alpha-enolase [32], which have demonstrated inhibitory activities against DPP-IV, ACE, and, in some cases, α-glucosidase [14]. For example, the antihypertensive activity attributed to dry-cured ham has been linked to the presence of peptides with the ability to inhibit the angiotensin-converting enzyme (ACE), such as AAPLAP, KPVAAP, and KAAAAP, which have shown relevant in vitro IC_50_ values [12,45] and hypotensive effects in animal models using spontaneously hypertensive rats (SHRs), where significant decreases in systolic blood pressure have been documented after administration of dry-cured ham extracts rich in these peptides [46]. On the other hand, the antioxidant action of soluble extracts from dry-cured ham has been linked to the presence of peptides such as SNAAC, GKFNV, GLAGA, or AEEEYPDL, which act through hydrogen transfer (HAT) and electron transfer (SET) mechanisms, contributing to the neutralization of free radicals and the prevention of oxidative damage in lipid systems and cellular models [47]. In the field of antidiabetic bioactivity, various peptides such as AEEEYPDL, LGVGG, GGLGP, and small dipeptide fragments (VE, PP, and EA) have shown the ability to inhibit the activity of DPP-IV and α-glucosidase [48], participating in the modulation of the postprandial glycemic response and suggesting a potential role in the control of type-2 diabetes [13].

Peptides with antimicrobial activity have also been identified, such as the pentapeptide RHGYM, which has shown efficacy against *Listeria monocytogenes* in food microbiology tests [49], representing an example of the potential use of dry-cured ham as a source of compounds with natural preservative properties. Finally, although less studied, peptides with possible anti-inflammatory activity have been reported, linked to the modulation of immune response mediators. Some in vitro studies and preliminary clinical trials have shown that regular consumption of dry-cured ham may contribute to improving the inflammatory profile and thrombogenic status in healthy subjects or those with cardiovascular risk factors [43,50].

All of this evidence positions dry-cured ham not only as a food with traditional and gastronomic value, but also as a matrix with high functional potential that can be exploited for the development of functional foods and nutraceuticals aimed at the prevention of chronic diseases.

### 3.1. Multifunctionality of Di- and Tripeptides Identified in Dry-Cured Ham

The di- and tripeptides generated during the maturation of dry-cured ham represent a significant fraction of the bioactive compounds present in this food. These small peptides are products of the sequential action of exopeptidases—such as dipeptidyl peptidases and tripeptidyl peptidases—and their small size gives them unique properties [51]: high solubility, greater relative resistance to gastrointestinal hydrolysis, and a higher probability of being absorbed intact through the intestinal epithelium, making them ideal candidates for exerting direct physiological effects. Several studies have identified di- and tripeptides with multifunctional activities, capable of acting on more than one biological target, such as inhibition of ACE, DPP-IV, and α-glucosidase, as well as antioxidant or glucose uptake modulating potential [32]. This multifunctional profile has been corroborated both by in vitro assays [52] and in silico studies [33,53], which highlight the frequency of these fragments in precursor proteins and their potential affinity for various enzymes and receptors. The main multifunctional di- and tripeptides identified in dry-cured ham are listed in Table 1 and Table 2.

These di- and tripeptides represent a peptide fraction of functional interest, not only because of their abundance and ease of intestinal absorption, but also because of their multifunctional capacity. More than 40 different dipeptides have been identified in dry-cured ham, mainly derived from structural proteins. Among the dipeptides identified, we highlight those with hydrophobic or aromatic residues in their C-terminal position, such as EA, VE, GP, KA, RG, PP, VK, or DL, which have demonstrated inhibitory capacity against angiotensin-converting enzyme (ACE) in both in vitro studies and animal models [45,46,64]. The dipeptide AA has been quantified and studied because it is one of the most abundant and has the most significant inhibitory capacity [54]. The presence of basic or branched residues also seems to improve or favor the interaction of dipeptides with the active site of ACE, as has been described for EK, VF, KP, and LA [14,52,55].

Conversely, numerous dipeptides have also been identified for their antidiabetic activity, acting as inhibitors of DPP-IV and, to a lesser extent, α-glucosidase. Notable examples are EA, VE, PP, PE, IP, II, LL, VF, SI, and SV, many of which are derived from proteins with high degradation rates during the maturation process [14,32,52,67,68]. The chemical structure and hydrophobic context of these dipeptides appear to play a crucial role in their affinity for DPP-IV, as evidenced by molecular docking models and in vitro validation [53,57]. For example, Gallego et al. (2014) identified di- and tripeptides with this activity in dry-cured hams, such as KA and GP, with IC_50_ ≈ 6–9 mol/L [52]. In addition, during gastrointestinal digestion, active fragments such as WT, KP, PL, and IP, which are believed to be responsible for DPP-IV inhibition in the intestine, could be released [52,78].

Antioxidant activity has also been documented in dipeptides such as AW, EL, AY, KP, and VY, with the ability to donate electrons or protons through SET and HAT mechanisms. This property is particularly interesting in the context of functional foods, as it contributes to cell protection against oxidative stress, which is involved in aging processes and chronic diseases [7,61]. Some dipeptides such as GA, VG, DG, DA, and EE have been associated with anti-inflammatory effects, showing the ability to modulate pro-inflammatory signaling pathways or inhibit mediators such as TNF-α, IL-6, and cyclooxygenase-2 (COX-2), according to enzymatic and cellular studies [18,61,62,64].

The dipeptides DA, DD, EE, ES, and LL showed the ability to reduce cholesterol synthesis by inhibiting 3-hydroxy-3-methylglutaryl-CoA reductase (HMG-CoA reductase). Through in vitro enzyme assays and in silico studies, Heres et al. (2021) confirmed this hypocholesterolemic capacity of dipeptides due to the in vitro enzymatic inhibition of HMG-CoA reductase (decrease in catalytic activity) and in silico models by molecular docking, demonstrating statin-like interactions with the active site of the enzyme [63].

It should be noted that some dipeptides such as EA, KP, GA, PP, and LA have clear multifunctionality, as they have been described simultaneously as antihypertensive, antidiabetic, antioxidant, and anti-inflammatory in different experimental models [14,32,52,55,61,64]. This functional convergence is particularly attractive for the design of nutraceuticals or broad-spectrum functional ingredients.

Tripeptides identified in dry-cured ham also have a high probability of intact absorption in the digestive tract and a bioactivity profile that is multifunctional in many cases (Table 2). Their structure allows for greater diversity of interactions with biological targets, and their generation is mainly associated with the action of tripeptidyl peptidases on degraded structural proteins such as titin, myosin, and troponin.

**Table 2:** Multifunctional tripeptides identified in dry-cured ham.

Sequence	Mass (Da)	Protein of Origin	Described Bioactivity	References
AAP	245.27	Proteolyzed Polypeptides/Light Myosin	Antihypertensive	[40,79]
AKK	345.44	Titin (fragments)	Antihypertensive	[7,32,71]
ALM	/	/	Antihypertensive	[80]
DVK	346.39	Ubiquitin	Antihypertensive	[40,79]
EAK	346.18	Titin (fragments)	Antioxidant	[7,32]
EEE	375.29	Ubiquitin	Antioxidant	[40]
EEL	/	/	Antihypertensive	[42]
EGV	275.28	LIM Domain-Binding Protein 3	Antioxidant	[40]
EKL	388.49	Myosin Light Chain	Immunomodulatory	[7,72,73]
ESV	/	/	Antihypertensive	[42]
LPK	343.43	Myosin	Antihypertensive	[79]
PAP	297.33	Titin	Antihypertensive	[7,32,71,79]
PFP	359.42	Myosin	Antidiabetic/Antiobesity	[14]
PPK	357.42	Titin/Myosin	Antihypertensive	[79]
SGL	275.28	Creatine Kinase	Antioxidant	[40,79]
SGP	259.26	Myosin Light Chain	Antihypertensive	[7,32,71]
SGV	261.29	Creatina quinasa	Antioxidant	[40,79]
STY	369.39	LIM Domain-Binding Protein 3	Antidiabetic	[32,52]
TNP	330.34	Myosin Light Chain	Antihypertensive	[7,32,71]
VAP	271.34	Myosin	Antihypertensive	[40,79]
VDY	/	/	Antidiabetic	[14]
VPL	271.34	Troponin T	Antihypertensive	[40,79]
YPG	335.35	Myosin	Antidiabetic/Antiobesity	[14]
YPL	391.46	Myosin	Antidiabetic/Antiobesity	[14]

For instance, AAP, PAP, PPK, LPK, VAP, VPL, DVK, SGP, and TNP have been identified as potent ACE inhibitors, many of them quantified directly by LC-MS/MS in dry-cured hams subjected to long maturation times [32,81]. Their efficacy is related to the presence of hydrophobic or acidic residues at the C-terminal position, as well as conformations that mimic the natural substrate of ACE [7,32,79]. Other peptide fragments have demonstrated the ability to neutralize free radicals and protect against lipid peroxidation [32,60,81,82,83,84]. These peptides, derived from creatine kinase and ubiquitin, have been shown to modulate the redox balance in cellular models and oxidized lipid systems [40]. Some in silico studies have also linked their activity to the density of electron-donating functional groups (−OH and −NH_2_) in the structure of the tripeptide [32].

Other tripeptides such as STY have been reported to have antidiabetic activity, inhibiting enzymes such as DPP-IV, especially when they contain aromatic residues (Tyr and Trp) that can be stabilized by interactions with the active site of the enzyme [32,52]. As with dipeptides, some tripeptides show remarkable multifunctionality. For example, VAP and VPL have not only been identified as ACE inhibitors, but also as antioxidants in cellular systems [85]. Fragments such as PAP, AKK, or EKL have been described as immunomodulators, implicating them in the regulation of inflammatory responses or neuronal functions, especially when derived from proteins such as titin or light myosin [7,72,73]. Mora et al. (2020) [14] identified and characterized three peptides with antiobesity activity—PFP, YPL, and YPG—all derived from dry-cured ham. These peptides not only showed inhibition of α-glucosidase and DPP-IV but also significantly reduced lipid accumulation in 3T3-L1 adipocytes, suggesting a potential antiadipogenic effect.

### 3.2. Angiotensin-Converting Enzyme (ACE) Inhibitory Peptides

Dry-cured ham is a food in which, due to its maturation process, endogenous proteolysis releases numerous peptides and free amino acids [4], several of which exhibit antihypertensive activity, mainly through the inhibition of angiotensin-converting enzyme (ACE) [45,54,61]. Table 3 presents a compilation of bioactive peptides (≥4 amino acids) reported in the literature for dry-cured ham with antihypertensive activity. Their sequences, molecular mass, precursor protein, type of experimental validation, antihypertensive activity (expressed as IC_50_ of ACE, % inhibition, or another equivalent measure), and the corresponding study reference are detailed.

As can be seen in Table 3, most of the antihypertensive peptides identified in dry-cured ham are 5–7 amino acids, with molecular masses of ~500–800 Da. Several peptides are derived from myofibrillar (muscle) proteins, such as myosin (AAPLAP and KPVAAP), titin (IAGRP, KPGRP, PSNPP, and TKYRVP), and myosin light chains, whereas others are derived from soluble muscle proteins (aspartate aminotransferase in TGLKP) and from connective tissue proteins such as elastin (GGVPGG) [9]. Peptides from less conventional proteins such as dinein (HCNKKYRSEM) [87] or metabolic enzymes such as allantoicase (AAATP) [9] have also been detected. These studies reflect the extensive proteolysis taking place during curing, which releases fragments of multiple muscle proteins [4,45].

The main mechanism of action of these peptides in lowering blood pressure is through the inhibition of the angiotensin-converting enzyme (ACE) [9]. ACE is a zinc-dependent metalloprotease that catalyzes the conversion of angiotensin I to angiotensin II, a potent vasoconstrictor; it also degrades bradykinin (vasodilator). Therefore, ACE inhibition produces a vasodilator effect and a decrease in blood pressure [88]. Many of the peptides identified act as competitive ACE inhibitors, binding to the active site of the enzyme and preventing substrate binding. ACE inhibitor peptides usually have hydrophobic, cyclic, or even aromatic amino acids at their C-terminal end, which interact strongly with the enzyme. In fact, it has been observed that the peptides with the best inhibitory potential in dry-cured ham contain proline (P) in the C-terminal (e.g., KPVAAP, AAPLAP, KAAAAP, and IAGRP), which is consistent with the preferences of ACE inhibitors. Many known inhibitors, such as the tripeptides IPP and VPP in milk, have a C-terminal proline [57]. In addition, several peptides contain a basic residue (Lys or Arg) in a position close to the C-terminus (e.g., Lys in the penultimate position in TGLKP or Arg in IAGRP). These positively charged amino acids could favor electrostatic interactions with the active site of ACE, which has negatively charged residues and zinc ions. The combination of an internal basic residue and a proline at the C-terminus appears to contribute to the high inhibitory affinity reported for bioactive peptides.

The number of amino acids contained in the peptide is also a factor to consider. Although it has been suggested that longer peptides may interact with more sites in ACE, this does not always translate into greater potency because they may be less stable and more prone to degradation [33,35]. In the case of dry-cured ham, peptides with 4–7 amino acids tend to show the lowest IC_50_ (i.e., the highest activity). For example, KPVAAP exhibits the lowest IC_50_ (~12 µM), followed by AAPLAP (6 aa, ~14 µM), whereas longer peptides such as TSNRYHSYPWG or FNMPLTIRITPGSKA showed significant inhibitory activity only at relatively high concentrations (170 µM for >70% inhibition). This suggests that long peptides may act after fragmentation into shorter active peptides during gastrointestinal digestion [86]. In this sense, many of the listed peptides are susceptible to degradation by digestive peptidases, generating di- and tripeptides that may also act as ACE inhibitors. In addition to ACE inhibition, some peptides may modulate other relevant targets in hypertensive pathophysiology. Other study evaluated the ability of peptide fractions from dry-cured ham bones to inhibit the endothelin-converting enzyme-1 (ECE-1) and platelet-activating factor-associated phospholipase A_2_ (PAF-AH) [55]. Some inhibitory activity was observed on ACE-1 (although it decreased after in vitro digestion), as well as marked inhibition of PAF-AH after simulated gastrointestinal digestion. ACE-1 generates endothelin, a potent vasoconstrictor; its inhibition by peptides could complement the effect of ACE inhibition. PAF-AH is involved in vascular inflammation; its inhibition could confer additional vasoprotective effects. These findings suggest that dry-cured ham peptides could have multiple cardioprotective effects, acting on several pathways.

In animal models, the impact is more evident. In a study conducted by Escudero et al. 2013, the synthesized pentapeptide AAATP (dose 30 mg/kg) was administered by gastric tube to SHRs (spontaneously hypertensive rats), and a marked reduction of –25.6 mmHg in systolic pressure was observed after 8 h [45]. Similarly, the peptides Ala-Ala (AA) and Ala-Trp (AW) present in dry-cured ham were recently evaluated in rats. Both achieved acute reductions in systolic pressure (~–20 mmHg) after oral administration [54,61]. However, peptides were rapidly metabolized; thus, their effect was transient. In contrast, 4–6 amino-acid-long peptides may have a slightly longer half-life, releasing active dipeptides gradually during gastrointestinal digestion [89].

A relevant question is whether dry-cured ham consumption has antihypertensive effects in humans. Traditionally, its consumption has been discouraged in hypertensive individuals due to its relatively high salt content; however, recent research suggests that the bioactive peptides present may partially counteract the hypertensive effect of sodium. A recent study with more than 13,000 Spanish adults followed for a period of 4.5 years found that high consumption of dry-cured ham (≥50 g at least 5 times/week) was not associated with a higher incidence of hypertension compared to low consumption (<1 time/week). These results indicated that, in a real dietary context, dry-cured ham did not have the expected harmful effect of contributing to high blood pressure. The authors suggested that the ACE-inhibiting peptides present in dry-cured ham could explain the impact on neutralization of the salt [90]. Controlled clinical trials have also been reported in the literature. Two studies carried out by Montoro-García et al., 2017, and Montoro-García et al., 2022, showed a trend towards reduced systolic and diastolic blood pressure [43,50], with the latter study showing, after 8 weeks of consumption, a significant decrease of ~2–3 mmHg in both systolic and diastolic pressure 24 h after ingestion of dry-cured ham, compared to the boiled ham control group. In addition, the latter study also observed a reduction in total blood cholesterol in the dry-cured ham group [43].

### 3.3. Antioxidant Peptides

During dry-cured ham maturation, endogenous proteolysis releases peptides that can act as antioxidants [39]. Table 4 shows the antioxidant peptides (>3 amino acids) identified in dry-cured ham. These peptides have been obtained either directly by protein hydrolysis during curing or by specific extraction and purification techniques from dry-cured hams. Their antioxidant activities have been validated mainly in vitro by free radical scavenging assays (DPPH and ABTS), ferric reducing power (FRAP), or oxygen radical absorbance capacity (ORAC), and even in cellular models of oxidative stress.

The results in the table show that multiple endogenous peptides in dry-cured ham exhibit strong antioxidant activity. AEEEYPDL showed high antioxidant power, even higher than that of reduced glutathione [98]. This peptide was quantified at 0.148 fg/g of cured ham using MRM mass spectrometry. The levels of peptides present in cured ham depend largely on the type of peptide and the technological processing conditions [39]. Similarly, the peptide SNAAC achieved almost 96% DPPH radical scavenging activity, positioning itself as one of the most potent antioxidant peptides described in the literature. However, in vitro digestion studies reveal that these long peptides can be degraded in the gastrointestinal tract, losing much of their original activity [9].

In Chinese dry-cured hams (e.g., Xuanwei), novel antioxidant peptides have been discovered using combined extraction and in silico screening strategies. In particular, the peptides DPLPPGWEIK and APPAAPPASGWPPTR showed the ability to protect skin cells (HaCaT) from UV-induced oxidative stress, increasing their cell viability and activating the cytoprotective Nrf2/Keap1 pathway [91,92], constituting the first evidence of dry-cured ham peptides capable of protecting human cells from oxidative damage caused by radiation. It should be noted that many of the most active antioxidant peptides share common structural motifs: they are small to moderate in size, often enriched in amino acids such as proline (P) and glycine (G) [91], and frequently contain residues with side chains capable of donating hydrogen or electrons or acid groups for chelating metals. These characteristics facilitate the neutralization of free radicals and the interruption of oxidative cascades [82,99].

### 3.4. Dipeptidyl Peptidase-IV Inhibitory Peptides

Several peptides generated in dry-cured ham have been shown to inhibit the enzyme dipeptidyl peptidase-IV (DPP-IV). This enzyme is responsible for the rapid degradation of incretin hormones such as glucose-dependent insulinotropic peptide (GIP) and glucagon-like peptide type-1 (GLP-1), both of which are involved in stimulating postprandial insulin secretion. Inhibition of DPP-IV prolongs the half-life of these hormones, facilitating better blood glucose control, which justifies its consideration as a therapeutic target in the treatment of type-2 diabetes mellitus [100].

Given that the most effective peptides against DPP-IV tend to be very short, dipeptides or tripeptides with proline or alanine in the penultimate residue have not yet been identified or corroborated in dry-cured ham. In this context, several peptides generated during the curing process of dry-cured ham have shown inhibitory potential against this enzyme. Specifically, it has been reported that the sequences AAAAG and AAATP, extracted from Spanish dry-cured ham, exhibit DPP-IV inhibitory activity, with IC_50_ values between 6.3 and 9.7 mol/L, indicating a moderate affinity for the enzyme [52]. Similarly, in a 2021 study, two peptides (WTIAVPGPPHS and FKRPPL) were identified after hydrolysis of dry-cured pork loin with pepsin and pancreatin. These two peptides were the most promising DPP-IV inhibitors. Using in silico analysis, the authors detected sequences with abundant motifs recognized by BIOPEP as DPP-IV inhibitors [78].

### 3.5. Anti-Inflammatory Peptides

Research on anti-inflammatory peptides in dry-cured ham is still in early stages, but recent advances indicate great potential. Unlike antioxidant and DPP-IV inhibitor peptides, in the anti-inflammatory field, much of the evidence comes from the effect of peptide mixtures from dry-cured ham in biological models. For instance, a peptide extract from Xuanwei dry-cured ham was shown to significantly reduce the production of inflammatory mediators (in vitro and in vivo). In LPS-stimulated RAW264.7 macrophages, it inhibited the secretion of nitric oxide (NO) and pro-inflammatory cytokines (TNF-α and IL-6) [94,101], whereas in a mouse model of colitis, it attenuated weight loss, inflammatory infiltration in the colon, and the expression of COX-2, among other markers [94]. These observations suggest that the peptides generated in dry-cured ham could exert systemic anti-inflammatory effects. In another study, Gallego et al. (2019) [55] evaluated the anti-inflammatory activity of peptides isolated from Spanish dry-cured ham by inhibiting platelet-activating factor acetylhydrolase (PAF-AH), autotaxin (ATX), and lipoxygenase (LOX). PAF-AH activity was inhibited by up to 26.06% with a fraction containing 19 peptides, with FNMPLTIRITPGSKA being identified as the most active peptide. ATX was inhibited by up to 57.49% with a fraction containing 13 peptides, with the strongest inhibition being that of the PSNPP pentapeptide, and LOX was inhibited by up to 23.33% with a fraction of 5 peptides, with HCNKKYRSEM showing the strongest inhibitory activity.

A recent study identified GPTGF, along with other peptides derived from the hydrolysis of collagen from ham bones, as potential anti-inflammatory agents. In vitro tests showed that bone peptide hydrolysate (in which GPTGF is one of the main components) suppresses the release of IL-6 and TNF-α in activated macrophages, suggesting that GPTGF and similar peptides could reduce the pro-inflammatory polarization of macrophages and cytokine production [101].

### 3.6. Other Bioactivities Associated with Peptides Identified in Dry-Cured Ham

Besides the antioxidant, antihypertensive, anti-inflammatory, and antidiabetic properties commonly reported in bioactive peptides derived from dry-cured ham, recent research has identified a number of complementary bioactivities of functional interest.

The fermentation process of dry-cured ham can cause protein degradation through the combined action of endogenous enzymes and microbial activity, resulting in higher concentrations of bioactive peptides [91,102]. Several peptides generated during the proteolysis of dry-cured ham have demonstrated potent activity against some food pathogens. In particular, the pentapeptide RHGYM was isolated from Spanish dry-cured ham and demonstrated a great ability to inhibit the growth of *Listeria monocytogenes* (MIC = 6.25 mol/L), being the most effective of those identified by Castellano et al. 2016 [49]. Other peptides, such as HCNKKYRSEM and MDPKYR, which contain the central KYR sequence with recognized antimicrobial activity, also showed efficacy, although at higher concentrations (MIC ≈ 50 mol/L). Similar results were obtained when three peptide fractions were extracted, namely QYYNG EEHVRFDSDVGEYR, LRNLPNLEVLDLGTNFI, and FASFEAQGALAN IAVDK from Chinese Xuanwei dry-cured ham, which produced a significant inhibition of the growth of *Escherichia coli* O157:H7 [7].

## 4. Use of Bioactive Peptides Derived from Dry-Cured Ham as Potential Nutraceuticals

The current trend toward developing foods with beneficial properties has opened up the possibility of using bioactive peptides present in dry-cured ham as nutraceutical ingredients or in the design of functional foods. Various studies have shown that these peptides can have beneficial effects on human health [43,50,90,103], suggesting its potential preventive or adjuvant application towards chronic diseases. For the development of nutraceuticals, concentrated sources of active peptides are necessary. In the case of dry-cured ham, methods for extracting the soluble peptide fractions after maturation have been tested, followed by separation techniques such as ultrafiltration or chromatography, to isolate the peptides of interest [46,104]. These fractions constitute the fundamental raw material for the development of nutraceuticals and enriched functional foods. A crucial step is demonstrating that peptides retain their bioactivity when administered in biological models. Numerous peptides isolated from dry-cured ham have shown activity using in vitro systems and animal models [95,98]. Peptide extracts from dry-cured ham have also exhibited anti-inflammatory effects, attenuating the production of pro-inflammatory cytokines [94,101]. This evidence is supported by clinical trials that have been conducted, which have achieved a significant decrease in blood pressure [43].

Bioactive peptides in dry-cured ham could be incorporated into different formats containing a standardized dose of peptides with defined bioactivity. Another possibility could be to fortify foods with these peptides [46,102]. Similarly, fat-free soups or broths made from dry-cured ham, concentrated in peptides, could be developed to act as functional broths, providing physiological benefits [105,106]. An important technological challenge is to ensure the stability of the peptides during product processing and storage, protecting them from thermal or pH degradation.

**Figure 3:**
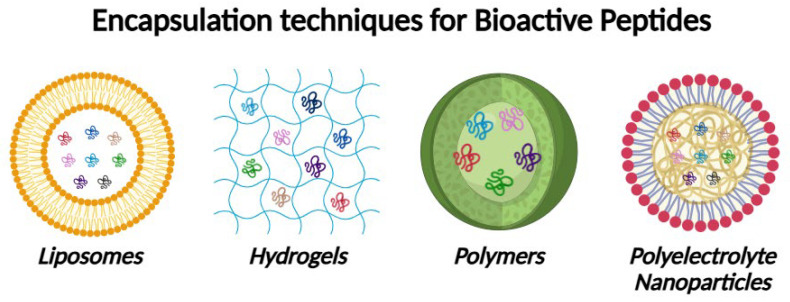
Techniques used for the encapsulation of bioactive peptides. Created with BioRender.com (accessed on 10 August 2025).

In this regard, techniques such as microencapsulation in polymer or lipid matrices have been successful in protecting bioactive peptides from degradation and releasing them in a controlled manner in the gastrointestinal tract. Recent studies describe the nanoencapsulation of peptides in liposomes and polyelectrolyte nanoparticles, which improves their gastric stability and intestinal absorption [107,108,109]. In the Figure 3 it could be seen the different techniques used for the encapsulation of bioactive peptides.

Such approaches could be applied to dry-cured ham peptides to develop more effective nutraceuticals. As with any nutraceutical ingredient, it is essential to ensure the safety of dry-cured ham peptides, although, as they are derived from a traditional food, these peptides have no known intrinsic toxicity; in fact, regular consumption of dry-cured ham in moderate amounts has been associated with neutral or beneficial effects on blood pressure and lipids in humans [90]. However, concentrating these peptides in a supplement may require further evaluation. Fortunately, there are already precedents for food peptides approved in nutraceuticals [110,111,112].

The nutraceutical application of peptides derived from dry-cured ham is a promising field. Different peptides with well-defined activities could be incorporated into supplements or functional foods aimed at the general population or at-risk groups. Before commercialization, it will be essential to demonstrate their efficacy in clinical trials and optimize formulations to ensure the release and bioavailability of these peptides in the body. With advances in research and technological innovation, it is not inconceivable that dry-cured ham—or its peptide extracts—will also become an important element in nutraceutical strategies for health promotion in the near future.

## 5. Conclusions and Future Directions

The bioactive peptides identified in dry-cured ham represent an interesting and rapidly evolving field of study. Current evidence suggests that dry-cured ham, beyond its culinary value, provides molecules with functional potential that could help prevent or mitigate risk factors for various chronic diseases. In particular, peptides have been characterized to have the ability to modulate cardiovascular function (by inhibiting the angiotensin-converting enzyme, blocking the endothelin axis, or inhibiting the PAF-AH factor), improve cellular oxidative balance (by scavenging free radicals and increasing the bioavailability of nitric oxide), and regulate glycemic metabolism (by inhibiting enzymes such as DPP-IV and α-glucosidase). Recent studies also highlight the multifunctionality of many of these peptides, where short sequences such as AA, VE, or KP simultaneously exhibit antihypertensive, antioxidant, and antidiabetic activities. This multifunctional profile is particularly interesting, as it suggests a synergistic or comprehensive effect on several physiological pathways, which could be exploited to address complex conditions such as metabolic syndrome through dietary interventions.

While some human studies have shown encouraging results, additional controlled clinical trials are needed. These studies should more directly evaluate the impact of dry-cured ham or its isolated peptides on clinical markers such as blood pressure, lipid profile, glycemic control, inflammatory status, etc. Long-term interventions in specific populations are of particular interest to confirm whether the incorporation of dry-cured ham into a balanced diet can have a protective or therapeutic effect. Recent clinical trials with dry-cured ham indicated a slight decrease of ~2–3 mmHg in blood pressure after several weeks of consumption, a clinically relevant result at the population level. Therefore, confirming and expanding these findings is a priority.

The conditions under which dry-cured ham is produced also influence the amount and type of peptides generated. Recent studies have shown that strategies such as prolonging maturation or slightly increasing the temperature in reduced-salt dry-cured hams can increase proteolysis and thus the release of bioactive peptides. These future lines of research point to the possibility of designing healthier versions of dry-cured hams by modulating the technological process. However, these changes must be balanced with the sensory quality of the dry-cured hams. Proteomics applied to different processing conditions is a valuable tool for identifying which practices optimize the generation of specific peptides without compromising safety or flavor. In the future, the use of proteolytic microbial starters or technological adjuvants that enhance the formation of certain functional peptides during curing could also be explored.

A promising direction is the valorization of dry-cured ham by-products as an additional source of peptides. For example, collagen peptides with notable anti-inflammatory activity, such as the pentapeptide GPTGF, have been isolated from dry-cured ham bones. The extraction of these peptides from by-products could lead to new functional ingredients, contributing to the sustainability of the industry. At the same time, the formulation of nutraceuticals based on dry-cured ham peptides is a field ready to be explored. In the coming years, we are likely to see dietary supplements with bioactive peptides aimed at controlling hypertension or improving metabolic health.

The mechanisms of action of many of these peptides in vivo are not yet known in detail. Further investigation into how they interact with their molecular targets will help understand their real therapeutic scope. Research into these interactions will open up new potential applications such as neurological health and appetite control. It is also important to evaluate the matrix effect within the ham itself, as the absorption of peptides may be affected by other components (e.g., fats and salt). Simulated gastrointestinal digestion and absorption studies in cell cultures will continue to be useful in determining what fraction of dry-cured ham peptides reaches the blood circulation in intact form, thus optimizing the consumption patterns.

Recently, technological advances have significantly expanded the set of tools available for discovering and characterizing bioactive peptides. In silico screening platforms, which integrate protein sequence databases (UniProt and BIOPEP-UWM) with predictive algorithms such as PeptideRanker, have become an indispensable tool for rapidly identifying candidate peptides with bioactive potential before conducting more costly in vitro or in vivo assays [58]. These bioinformatics tools can also simulate gastrointestinal digestion and predict the release of active fragments, guiding the selection of enzymes for hydrolysis [113].

Another new approach that has recently been used is the integration of peptidomics with molecular docking and molecular dynamics simulations to predict the interaction of peptides with specific molecular targets, such as ACE, DPP-IV, or α-glucosidase. Recent studies have shown that combining LC-MS/MS-based peptidomics with in silico docking not only allows for highly reliable identification of peptide sequences, but also provides mechanistic information on binding modes, stability, and potential synergistic effects [114]. This computational experimental process can accelerate the elucidation of multifunctional peptides from complex matrices such as cured ham, supporting their use in functional foods and nutraceutical formulations [113,115].

Finally, the convergence of advanced analytical chemistry, big data, and simulation is expected to transform future research in this field. Future studies should integrate omics datasets to better understand the matrix effects and systemic impact of cured ham peptides, combining these approaches with well-designed clinical trials to validate their functional potential. This integrative strategy will be essential to translate the growing mechanistic evidence into practical dietary recommendations, functional product development, and nutraceutical formulations. In this context, dry-cured ham has gone from being considered only a traditional product to being recognized as a food with functional potential thanks to the peptides liberated during its maturation. In the coming decades, there is likely to be an increase in research aimed at applying this knowledge in innovative ways, from the development of healthier meat products to the creation of specific dietary supplements, allowing cured ham, backed by a solid scientific basis, to consolidate its position not only as part of gastronomic culture, but also as a valuable ally in promoting health.

## Figures and Tables

**Table 1:** Multifunctional dipeptides identified in dry-cured ham.

Sequence	Mass (Da)	Protein of Origin	Described Bioactivity	References
AA	204.23	Myosin7590	Antihypertensive/Antidiabetic	[32,52,54,55,56]
AD	204.18	Myosin Light Chain	Antidiabetic	[14,57]
AL	188.21	Myosin	Immunomodulatory/Antidiabetic	[10,16,18,52,58]
AS	176.08	Actin	Antidiabetic	[59,60]
AW	246.3	Myosin	Antioxidant/Antihypertensive	[54,61]
AY	252.27	LIM Domain-Binding Protein 3	Antioxidant	[32]
DA	204.18	Actin/Myosin	Anti-inflammatory/Antihypertensive/Lipid-Metabolism-Modulating Activity	[18,33,61,62,63]
DG	190.15	Myosin	Anti-inflammatory/Antihypertensive/Antioxidant	[33,61,62,64]
DL	246.28	Creatine Kinase	Antihypertensive	[55]
EA	218.21	Titin/Myoglobin	Antihypertensive/Antidiabetic	[14,52,55,62,65]
EE	276.24	Titin (fragments)	Anti-inflammatory/Antihypertensive	[33,61,64,66]
EF	278.35	Ubiquitin-60S	Antihypertensive	[32,67]
EK	275.3	Myosin	Antihypertensive/Antidiabetic	[55]
EL	244.3	Enolase	Antioxidant/Antihypertensive	[7,32,42]
ES	234.21	Titin	Anti-inflammatory/Antihypertensive/Antioxidant	[33,61,62,64]
EV	277.24	Myosin	Antyhypertensive	[42,58]
EW	333.34	Myosin	Antihypertensive	[14]
GA	146.15	Titin	Anti-inflammatory/Antidiabetic/Antihypertensive	[14,18,32,33,53,55,61,62,63,64]
GP	172.2	Titin	Antihypertensive/Antidiabetic	[32,52,55,61,64]
IE	260.29	α-enolase	Antihypertensive/Antidiabetic	[14,33,68]
IF	278.35	Ubiquitin-60S	Antihypertensive	[14]
II	244.33	Glyceraldehyde-3-Phosphate Dehydrogenase	Antidiabetic	[32,67,69]
IL	244.33	Enolase	Antidiabetic	[32,67,69]
IP	244.3	Troponin T	Antidiabetic	[32]
IV	230.3	Myosin	Antidiabetic	[32,67,69]
KA	217.27	Ubiquitin-60S	Antihypertensive/Antidiabetic Lipid-Metabolism-Modulating Activity	[7,32,52,55,56,70,71]
KP	243.3	Titina	Antidiabetic/Antihypertensive Antioxidant	[7,32,55]
LA	202.25	Creatine Kinase	Antihypertensive/Antidiabetic	[55]
LI	244.33	Myosin	Antidiabetic	[7,32,69]
LL	244.33	Lactate Dehydrogenase	Antidiabetic/Immunomodulatory	[7,32,67,69,72,73]
LR	287.36	Myosin Light Chain	Antihypertensive Brain Health-Promoting and Neuron-Related Activities	[7,10,32,40,56,67]
NR	289.29	Creatine Kinase	Antihypertensive	[32,67]
PE	244.25	Titin/Myosin	Antidiabetic	[14,74]
PG	172.18	Actin	Antidiabetic	[32,53,61]
PL	228.3	Creatine Kinase	Antidiabetic/Antihypertensive	[14,52,55]
PP	212.25	Myosin	Antihypertensive/Antidiabetic	[14,55,66,68,75]
QN	260.25	Myosin	Antidiabetic	[59,60]
RG	246.28	Troponin T	Antihypertensive/Antidiabetic	[55]
RL	287.36	LIM Domain-Binding Protein 3	Antihypertensive/Immunomodulatory	[7,42,72,73]
RP	271.32	Myosin	Antihypertensive	[32,55,56]
SI	246.3	α-enolasa	Antidiabetic	[14,57]
SV	232.3	Titin	Antidiabetic	[32]
VD	232.23		Antidiabetic	[14,33]
VE	246.26	Titin	Antihypertensive/Antidiabetic	[14,55,57,66,68]
VF	262.30	Myosin Light Chain	Antihypertensive/Antidiabetic	[14,18,58,63,64]
VG	174.20	Myosin	Anti-inflammatory/Antihypertensive/Immunomodulatory	[33,61,62]
VH	254.29	Troponin T	Immunomodulatory	[10,16,18,52]
VK	245.32	Myosin Light Chain	Lipid-Metabolism-Modulating Activity	[7,55,70,71]
VV	216.28	LIM Domain-Binding Protein 3; β-enolasa	Antidiabetic	[14,76]
VY	280.32	Enolase	Antihypertensive/Antioxidant Brain Health-Promoting and Neuron-Related Activities	[7,10,32,40,55,56]
WK	319.4	β-enolase	Antidiabetic	[14,76]
YA	252.27	Myosin	Antihypertensive/Antidiabetic	[59,60,77]

**Table 3:** Peptides identified in dry-cured ham with antihypertensive activity.

Sequence	Mass (Da)	Protein of Origin	Validation Methodology	Antihypertensive Activity	Reference
AAATP	429.5	Allantoicase (metabolic enzyme)	In vitro (ACE inhibition), in vivo (SHR* rat)	IC_50_ = 100 µM PAS* decrease = –25.6 mmHg (8 h)	[45]
AAPLAP	538.6	Heavy chain of myosin XV	In vitro (ACE inhibition)	IC_50_ = 14.38 µM	[86]
AMNPP	528.6	Myosin 3 (heavy chain)	In vitro (ACE inhibition)	IC_50_ = 304.5 µM	[86]
ASGPINFT	805.9	Myosin regulatory light chain 2	In vitro (ACE inhibition)	IC_50_ = 975 µM	[45]
DVITGA	574.6	Light chain of myosin	In vitro (ACE inhibition)	IC_50_ = 900 µM	[45]
FNMPLTIRITPGSKA	1646.0	Fragment of myofibrillar protein	In vitro (ACE inhibition)	>70% inhibition at 170 µM	[55]
GGVPGG	442.5	Elastin	In vitro (ACE inhibition)	79.9% inhibition at 1 mol/L	[55]
GVVPL	483.6	Heavy chain of myosin	In vitro (ACE inhibition)	IC_50_ = 956 µM	[80]
HCNKKYRSEM	1295.5	Heavy chain of dynein 3	In vitro (ACE inhibition)	>70% inhibition at 170 µM	[55]
IAGRP	512.6	Titin (myofibrillar protein)	In vitro (ACE inhibition)	IC_50_ = 25.94 µM	[86]
IKLPP	566.7	Myosin IXb (heavy chain)	In vitro (ACE inhibition)	IC_50_ = 193.9 µM	[86]
KAAAAP	527.6	Light chain 3 of myosin	In vitro (ACE inhibition)	IC_50_ = 19.79 µM	[86]
KAAAATP	628.7	PR-domain zinc finger protein 2	In vitro (ACE inhibition)	IC_50_ = 25.64 µM	[86]
KPGRP	553.7	Titin	In vitro (ACE inhibition)	IC_50_ = 67.08 µM	[86]
KPVAAP	581.7	Myosin	In vitro (ACE inhibition)	IC_50_ = 12.37 µM	[86]
KVLPG	512.7	Phosphoglycerate kinase 1	In vitro (ACE inhibition)	IC_50_ = 265.4 µM	[86]
PAPPK	508.6	Light chain 1/3 of myosin	In vitro (ACE inhibition)	IC_50_ = 199.6 µM	[86]
PSNPP	510.5	Titin	In vitro (ACE inhibition)	IC_50_ = 192.8 µM	[86]
SFVTT	553.6	Glyceraldehyde-3-phosphate dehydrogenase	In vitro (ACE inhibition)	IC_50_ = 395 µM	[80]
TGLKP	514.6	Aspartate aminotransferase	In vitro (ACE inhibition)	IC_50_ = 51.57 µM	[86]
TKYRVP	762.9	Titin	In vitro (ACE inhibition)	>70% inhibition at 170 µM	[55]
TSNRYHSYPWG	1367.4	Protein kinase	In vitro (ACE inhibition)	>70% inhibition at 170 µM	[55]

* PAS = systolic blood pressure; SHRs = spontaneously hypertensive rats. In the activity column, the inhibition percentages correspond to the concentration indicated when no IC_50_ was reported.

**Table 4:** Peptides with antioxidant activity identified in dry-cured ham.

Sequence	Mass (Da)	Protein of Origin	Validation Methodology	Antioxidant Activity	Reference
AEEEYPDL	1109	Creatine kinase (muscle)	In vitro (ABTS capture, ORAC)	ABTS: 1474 nmol TEAC/mg; ORAC: 960 nmol TE/mg (high antioxidant activity)	[39]
APPAAPPASGWPPTR	1743	/	Cell model (HaCaT + UVA)	Protected HaCaT cells from oxidative damage caused by UVA, increasing their survival and antioxidant response	[91,92]
APYMM	611.76	/	In vitro (ABTS)	ABTS: 0.12 mg/mL	[93]
DLEE	576	/	In vitro (ABTS, ORAC); cell model (Caco-2)	ABTS: 148 μmol TE/g; ORAC: 1032 μmol TE/g (similar to glutathione); reduced intracellular ROS and activated the Nrf2-Keap1 pathway	[94]
DPLPPGWEIK	1331	E3 ubiquitin ligase	Cell model (HaCaT keratinocytes + UVA)	Increased the viability of UVA-damaged HaCaT cells (~10–15% vs. control), activating the Nrf2-Keap1 antioxidant pathway	[91,92]
FLKMN	652	Myosin light chain	In vitro (DPPH)	DPPH: 65% inhibition at 1 mg/mL; OH−:60% at 1 mg/mL	[95]
FWIEE	706.84	/	In vitro (ABTS)	ABTS: 0.23 mg/mL	[93]
GKFNV	564	/	In vitro (DPPH)	DPPH: 92.7% inhibition at 1 mg/mL	[47,95]
LPGGGHGDL	822	/	In vitro (O^-^)	OH−: 85% at 1 mg/mL	[95]
LPGGGT	501	/	In vitro (DPPH)	DPPH: 65% inhibition at 1 mg/mL; OH−: 60% at 1 mg/mL	[95]
MWTD	551.61	/	In vitro (ABTS)	ABTS: 0.4 mg/mL	[93]
SAGNPN	640	Integrin α-3 (muscle)	In vitro (DPPH)	DPPH: 50% inhibition at 1.5 mg/mL (moderate antioxidant activity)	[12]
SNAAC	555	Heavy myosin chain	In vitro (DPPH, reducing power)	DPPH: 95.7% at 3 mg/mL; FRAP: Abs_700 = 1.7 at 1 mg/mL (potent antioxidant)	[96,97]

TE = Trolox Equivalent; TEAC = antioxidant capacity equivalent to Trolox. DPPH: % radical inhibition; FRAP: absorbance at 700 nm (reducing power).

## Data Availability

Not applicable.
